# A Possible Novel Protective Effect of Piceatannol against Isoproterenol (ISO)-Induced Histopathological, Histochemical, and Immunohistochemical Changes in Male Wistar Rats

**DOI:** 10.3390/cimb44060171

**Published:** 2022-05-27

**Authors:** Samar A. Alghamdi, Maryam H. Mugri, Nahid M. H. Elamin, Mona Awad Kamil, Hind Osman, Basma G. Eid, Rasheed A. Shaik, Soad S. Shaker, Aziza Alrafiah

**Affiliations:** 1 Department of Oral Biology, Faculty of Dentistry, King AbdulAziz University, Jeddah 22254, Saudi Arabia; samalgamdi@kau.edu.sa; 2 Department of Maxillofacial Surgery and Diagnostic Sciences, College of Dentistry, Jazan University, Jazan 45142, Saudi Arabia; dr.mugri@gmail.com (M.H.M.); nahide99@gmail.com (N.M.H.E.); 3 Department of Preventive Dental Science, College of Dentistry, Jazan University, Jazan 45142, Saudi Arabia; munakamil@yahoo.com (M.A.K.); hindalish6@gmail.com (H.O.); 4 Department of Pharmacology and Toxicology, Faculty of Pharmacy, King AbdulAziz University, Jeddah 22254, Saudi Arabia; beid@kau.edu.sa (B.G.E.); rashaikh1@kau.edu.sa (R.A.S.); 5 Department of Histology, Faculty of Medicine, Assiut University, Assiut 71515, Egypt; soadshaker@gmail.com; 6 Department of Medical Laboratory Sciences, Faculty of Applied Medical Sciences, King AbdulAziz University, Jeddah 22254, Saudi Arabia

**Keywords:** Ki-67, salivary gland, piceatannol, isoproterenol, histological, histochemical

## Abstract

Dry mouth is characterized by lower saliva production and changes in saliva composition. In patients with some salivary gland function remaining, pharmaceutical treatments are not recommended; therefore, new, more effective methods of promoting saliva production are needed. Hence, this study aimed to provide an overview of the histological changes in the salivary gland in the model of isoproterenol (ISO)-induced degenerative changes in male Wistar rats and to evaluate the protective effect of piceatannol. Thirty-two male Wistar rats were randomly divided into four groups: the control group, the ISO group, and the piceatannol (PIC)-1, and -2 groups. After the third day of the experiment, Iso (0.8 mg/100 g) was injected intraperitoneally (IP) twice daily into the animals. PIC was given IP in different daily doses (20 and 40 mg/kg) for three days before ISO and seven days with ISO injection. The salivary glands were rapidly dissected and processed for histological, histochemical, immunohistochemical (Ki-67), and morphometric analysis. Upon seven days of treatment with ISO, marked hypertrophy was observed, along with an increased number of positive Ki-67 cells. Proliferation was increased in some endothelial cells as well as in ducts themselves. Despite the significant decrease in proliferation activity, the control group did not return to the usual activity level after treatment with low-dose PIC. Treatment with a high dose of PIC reduced proliferative activity to the point where it was substantially identical to the results seen in the control group. An ISO-driven xerostomia model showed a novel protective effect of piceatannol. A new era of regenerative medicine is dawning around PIC’s promising role.

## 1. Introduction

The three major salivary glands are the submandibular glands, the parotid glands, and the sublingual glands. They generate much saliva every day, containing various nutrients to keep the digestive and dental systems healthy and crucial in identifying disease. To keep the mouth and throat healthy, the saliva produced by the salivary glands is essential [1].

Dry mouth is a disorder that occurs due to insufficient saliva secretion or absolute salivary gland dysfunction [2,3]. As a result of its effects on salivary function, dysphagia, digestion, taste, and appearance, a dry mouth can harm social interactions and interpersonal relationships. It may thus lead to the development of diseases such as dental caries (tooth decay), periodontitis (gum disease), halitosis (bad breath), and dental hypersensitivity. In addition, many health concerns, such as recurring dental cavities, mucosal infection, altered taste perception, and difficulties swallowing and speaking, can be caused by a lack of salivary secretions. As a result of these issues, many people suffer from chronic stress and despair [4,5,6]. Dry mouth is referred to in medicine as xerostomia, and it refers to the patient’s subjective experience of it [7].

Drugs are the most common cause of dry mouth. Salivary glands are susceptible to strict anticholinergics (atropine, belladonna, scopolamine, etc.). Other pharmacological groups which also produce xerostomia are antidepressants and antipsychotics (e.g., serotonin reuptake inhibitors, tricyclic and heterocyclic antidepressants), antihypertensives (ACE inhibitors, diuretics, beta-blockers, etc.), anxiolytics and sedatives, muscle relaxants, analgesics (CNS/opioids), antihistamines, appetite suppressants, acne medications, anticonvulsants, antiparkinson agents, bronchodilators, migraine medications, and hypnotics [8,9,10,11]. Type 1 diabetes, hyperthyroidism, renal failure, vitamin deficiencies, and some acute or chronic viral infections such as mumps, HIV and CMV, and various autoimmune connective tissue diseases are some of the other causes of dry mouth. Sjögren’s syndrome is the most prominent chronic condition, which is an autoimmune inflammatory condition that causes xerostomia, as a result of infiltration of salivary and lacrimal glands, and xerostomia is also a common condition associated with radiation therapy to the head and neck for the treatment of cancer, often as a result of injury to the salivary glands resulting in reduced salivary output [12]. As a result, patients regularly complain about having a dry mouth. In recent months, following the outbreak of the new coronavirus pandemic, some cases of dry mouth related to severe acute respiratory syndrome-coronavirus-2 (COVID-19) have been reported, which has attracted the attention of researchers [13].

Many types of receptors, including ß adrenoceptors, exist in salivary gland tissues, suggesting that salivary glands may contain target systems for many drugs such as β- receptor drugs [14,15]. Non-specific agonists such as isoproterenol (ISO), a non-selective type adrenoceptors agonist, have been widely used to study the mechanisms for adrenoceptors signaling in cellular immune response, including cardiomyocytes and adipocytes. Low levels of the concentrations trigger off-target effects [16]. Due to a particular adrenergic stimulation, ISO can suppress saliva production and promote the release of specific proteins and the most abundant are proline-rich proteins [17,18]. Researchers use ISO to understand adaptive stress mechanisms. The intraperitoneal or intravenous administration of isoproterenol (β-adrenergic agonist) reduces saliva’s release but markedly increases its protein content. In line with these findings, ISO increases the heart rate and enhances sinoatrial and atrioventricular conduction. However, in animal models, the chronic treatment with ISO causes severe stress to the cardiomyocytes, resulting in hypertrophy and myocardial infarct, like those observed in humans and closely related to oxidative stress and inflammation [17]. Based on the claim that isoproterenol cause angiotensin-converting enzyme (ACE2) overexpression in some animal experiments, concerns have arisen around the potential of ISO aggravating SARS-CoV-2 infection and coronavirus disease-2019 severity in isoproterenol-treated patients [19,20].

Each year, tens of thousands of people worldwide are affected by hypo-functioning salivary glands. However, pharmaceutical therapies for salivary dysfunction are few. Furthermore, the etiology of salivary gland injury is still unknown [21,22]. Some ailments are still untreatable by modern treatment, but natural remedies provide some solace. Unfortunately, many people use these natural remedies without realizing the consequences [23]. For various ailments, the World Health Organization (WHO) recommends testing the efficacy of plant medicines [24].

Putative therapeutic agents for metabolic diseases are phytochemicals, secondary plant metabolites formed in response to environmental stress. In the therapy of metabolic diseases, resveratrol is a well-researched phytochemical. However, one of the biggest obstacles to resveratrol’s efficacy in human trials is believed to be its low oral bioavailability [25]. To treat metabolic diseases, the development of stable resveratrol metabolites and synthetic derivatives with enhanced bioavailability is evolving [26].

In foods including grapes, blueberries, passion fruit seeds, and peanuts, piceatannol (PIC) is a polyhydroxylated stilbene [27]. PIC has an extra hydroxyl group attached to its 30-carbon backbone, unlike resveratrol. Evidence suggests that piceatannol is more metabolically stable in the body than resveratrol [28]. Hyperlipidemia, atherosclerosis, cardiovascular disease, and cancer are just a few piceatannol illnesses that can help [29]. The literature describes its anti-proliferative and invasive properties [30]. Piceatannol’s free radical scavenging activity has been demonstrated in the literature to be superior to that of resveratrol [31]. In addition, some researchers believe that PIC encourages anti-inflammatory signaling [27]. Several studies on resveratrol and salivary gland dysfunction are available for review [32,33,34]. When resveratrol was administered to NOD mice, for example, the malfunctioning of their salivary glands was alleviated. Using resveratrol as a preventative and therapeutic strategy against Sjögren’s syndrome has been suggested by the researchers [33].

Considering the protective effect of resveratrol in salivary gland dysfunction, we hypothesize that piceatannol may have a beneficial effect on salivary gland dysfunction. Based on this background, we aim to provide an overview of the histopathological changes in the salivary gland induced by isoproterenol in male Wistar rats and evaluate the potential protective effect of piceatannol.

## 2. Materials and Methods

### 2.1. Ethical Statement

Animal handling, medications, and scarification were carried out following the guidelines for the care of experimental animals and approved by the Research Ethical Committee, Faculty of Pharmacy, King Abdulaziz University, Saudi Arabia, according to the Animal Research Ethics Committee Guide for taking care and use of laboratory animals (Approval No. PH-134-41).

### 2.2. Chemicals

Isoproterenol (ISO) (0.8 mg/100 g body weight, Catalog no. I5627) was purchased from Sigma-Aldrich Inc. (St. Louis, MO, USA). Dimethyl sulfoxide (DMSO) and 0.9% saline was used to dissolve piceatannol (PIC) (2:3) (98% pure) supplied from Alexis Biochemicals (San Diego, CA, USA).

### 2.3. Animals

The animal house at the faculty of Pharmacy, King Abdulaziz University, Saudi Arabia, provided the male Wistar rats (weighing between 195 and 220 g) in the 10-week age range. The animals were housed in an air-conditioned environment (22.2 °C) with alternate light/dark cycles. The animals were fed a regular pellet diet and always given water access. Before beginning the experiment, the rodents were maintained in our facility for one week to acclimate them.

### 2.4. Experimental Design

Thirty-two male Wistar rats were randomly divided into four groups (*n* = 8 rats/group). Based on a pilot investigation, the dosages and timetables were determined. Different daily dosages of piceatannol (20 and 40 mg/kg) were administered just two hours before the injection of ISO.

The control (C) group received the vehicle (DMSO and isotonic saline) (2:3), 0.2 mL/200 gm body weight intraperitoneally (IP), once daily from day 0 for ten consecutive days and an injection of isotonic saline (0.2 mL/200 gm, IP) twice on the third day of the experiment for seven days.

The isoproterenol (ISO) group rats received vehicle IP, once daily for ten consecutive days and an injection of ISO (0.8 mg/100 g body weight) twice daily (morning and night) dissolved in isotonic saline from the third day of the experiment for seven days to induce the experimental xerostomia model [17,35].

The piceatannol-1 (PIC-1) group received PIC at a dose of 20 mg/kg [36], IP, once daily from day 0 for ten consecutive days and a twice-daily injection of ISO in the morning and again at night the third day of the experiment for seven days.

The piceatannol-2 (PIC-2) group received PIC at a dose of 40 mg/kg, IP, once daily from day 0 for ten consecutive days and a twice-daily injection of ISO in the morning and again at night on the third day of the experiment for seven days.

### 2.5. Histological Techniques

An intravenous dose of ketamine chloride and xylazine chloride (10 mg/kg) sedated the animals for 10 min before being killed. From the neck skin, fascia and ear base fascia were removed from the base of each ear and the caudal border of the mandibles. In addition, the major salivary glands (parotid, submandibular, and sublingual) were separately removed. Fixatives were used to preserve gland specimens, then subjected to histopathological, histochemical, and immunohistochemical analysis.

### 2.6. Histopathological Examination

The salivary gland specimens were fixed immediately in 10% formalin solution and then washed under running tap water to remove all fixative residues. Specimens were then dehydrated by being transferred in increasing concentrations of alcohol and then cleared by xylol. The dehydrated samples were then embedded in the center of paraffin wax blocks. The blocks were trimmed and cut into 5-μm thick sections. Sections were transferred in decreasing concentrations of alcohol and then distilled water to be stained with hematoxylin and eosin (HE) stains for routine histological examination [37]. A method for demonstrating connective tissue fibers using Masson’s Trichrome dye [38].

### 2.7. Histochemical Examination

For histochemical examination, transverse sections from the embedded tissues were cut and stained with Alcian blue 2.5 pH (AB 2.5 pH) for acidic mucin identification (acid mucopolysaccharides were stained blue) and counterstained with Nuclear Fast red using standard histological methods. Sections from the three salivary glands were stained with Periodic Acid-Schiff (PAS) for identification of neutral mucin (neutral mucopolysaccharides appear red) and counterstained with hematoxylin [39].

The slides were studied using a light microscope (Olympus binocular) (Model BX40F4, 7E12569) Olympus Optical co., LTD. Tokyo, Japan. at ×10 ×20 and ×40 magnifications. Photographs of the prepared slides mounted on the binocular microscope were digitally obtained using the Lecia ICC50 W camera. These pictures were transferred to a computer, and detailed studies were carried out. Relevant areas and structures were labeled.

### 2.8. Immunohistochemical Examination

Silane-coated glass slides were used to mount tissue sections of 4 µm thickness from formalin-fixed paraffin-embedded tissue blocks. Xylene was used to deparaffinized the paraffin sections. Water was used to rehydrate sections with decreasing alcohol concentrations. This procedure was carried out for antigen retrieval by heating a pressure cooker filled with sodium citrate buffer (0.01 M, pH 6.01). Phosphate-buffered saline (PBS; pH 7.4, 0.05 M) washed sections three times for 5 min each after peroxide block (3% hydrogen peroxide in water) incubation. For blocking nonspecific immune responses, the sections were incubated for 10 min at room temperature with a power block (a solution including casein and proprietary additives in PBS with 0.09% sodium azide). Mouse monoclonal antibody (anti-ki-67 antigen; cat. no. ab279653; Abcam; Cambridge, UK) was applied and incubated at room temperature (25 °C) for 60 min before being maintained overnight at 4 °C. Afterward, the section was counterstained with hematoxylin and eosin. Tissue size dictated how much super-enhancer reagent should be used to cover the specimen to maximize the antigen-antibody reaction’s potency (38 L). It was incubated for 30 min at room temperature (25 °C) with an appropriate volume of poly-horse radish peroxide reagent to cover the specimen and thoroughly washed with PBS three times. Next, the specimen was covered with a solution of 3,3′-diaminobenzidine (DAB) and incubated for 30 min at room temperature (25 °C). The specimen was then washed with PBS at least three times, and the specimen was examined. Using disterene polysterase xylene, the slides were counterstained with Mayer’s hematoxylin and placed on a coverslip [40]. Intranuclear brown color DAB staining in cells was obtained as evidence that they were positive for the Ki-67 antigen.

### 2.9. Morphometric Study

Both Masson trichrome stain and Ki-67 immuno-stained sections were examined using a light microscope (Model BX40F4, 7E12569) Olympus Optical co., LTD. Tokyo, Japan. Field photographing was carried out using a mounted camera (Olympus soft imaging solutions, Munster, Germany, Model LC20, 59001227). Five randomly selected, non-overlapping fields were inspected per five sections under × 40 magnification for the quantitative analyses. Images were then transferred to the computer system for analysis using image analysis software Image J (Image J, v1.41a, NIH, Bethesda, Maryland, USA) computer system, and then the obtained data were statistically analyzed. The area % (%) of collagen fibers was selected in the program and generated automatically for each image [41]. In addition, the number of Ki-67 positive nuclei was automatically counted.

### 2.10. Statistical Analysis

Statistical analysis was performed using SPSS (IBM SPSS Statistics 26.0, IBM Corporation, and Somers, NY, USA). Numerical data were explored for normality by checking the distribution of data and using tests of normality (Kolmogorov-Smirnov and Shapiro-Wilk tests). Data were normally distributed and had a homogeneity of variance. Values were determined as means ± standard deviation (SD). Comparison between groups was tested using one-way analysis of variance (ANOVA) followed by a Tukey’s Multiple Comparison Test (Post Hoc Tukey HSD). The significance level was set at *p* ≤ 0.05. GraphPad Prism version 9.0.0 for Windows, GraphPad Software, San Diego, CA, USA www.graphpad.com (accessed on 21 April 2022) were used to create the graphs.

## 3. Results

### 3.1. Histopathological Results

#### 3.1.1. Examination of Hematoxylin and Eosin-Stained Sections

**Figure 1:**
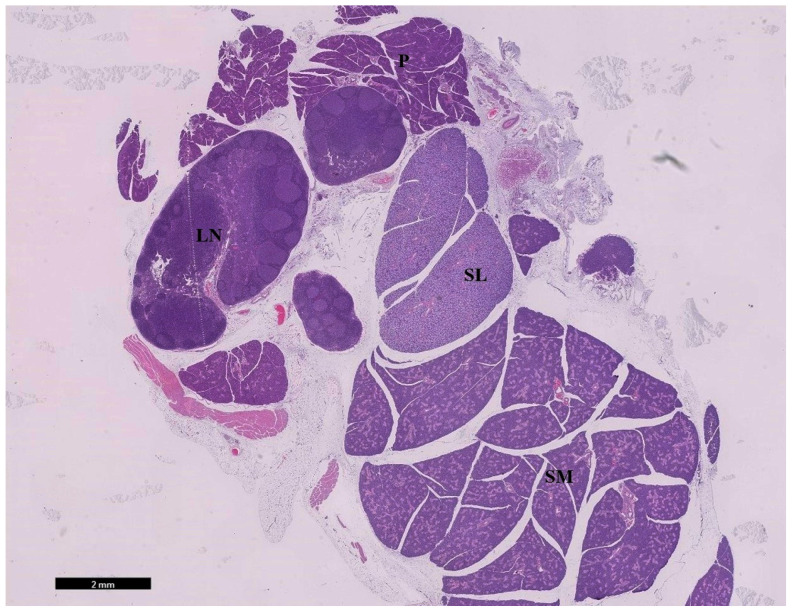
Photomicrographs of sections of rat major salivary glands from the control group showing salivary glands dissected with adjacent structures intact. Submandibular gland (SM), sublingual gland (SL), parotid gland (P), and mandibular lymph node (LN) (HE; ×5).

Salivary gland sections stained with HE showed close apposition between the three primary salivary glands and a part of the lymph nodes (submandibular, sublingual, and parotid glands). Each gland is protected by a thin fibrous capsule that merges into a larger capsule. In addition, connective tissue forms a thin stroma between the lobules of glands (Figure 1).

**The Parotid Gland**

**Figure 2:**
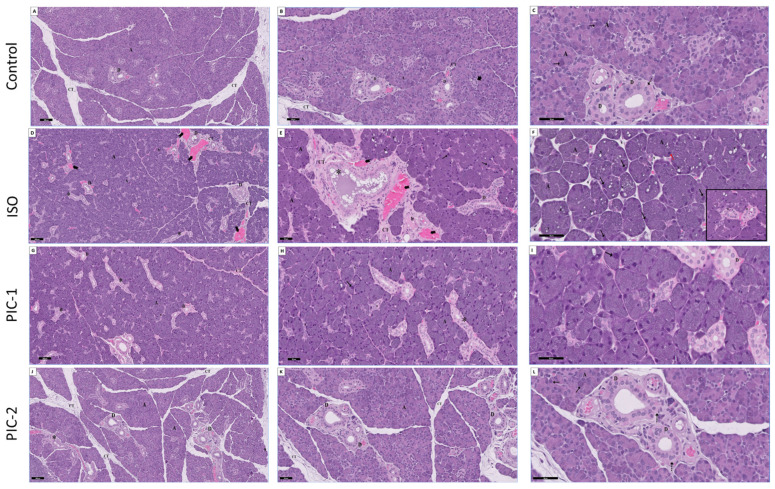
A section in the parotid gland stained with HE from the control group (**A**–**C**) showing the lobules of the gland, each lobule is formed of serous acini (A) and ducts (D) with wide connective tissue septa filled with a fine network of interlobular connective tissue fibers (CT) serous acini (A) consists of a single layer of pyramidal cells (↑) with basal rounded nuclei surrounding a lumen. Among the acini, intercalated ducts lined by cuboidal cells with rounded nuclei (arrowhead) and striated ducts (D) are observed and lined by a single layer of columnar epithelium (dot arrow) (HE; A × 100, B × 200, C × 400). ISO group (**D**–**F**) showing markedly hypertrophic packed secretory serous acini (A). Some of the acini exhibited irregular outline and spaces (s) appear between acini. The cytoplasm and basal lines of acinar cells are blurred, and the vacuolization (V) of acinar cells is obvious. Nuclei of serous acini appear enlarged and irregular in shapes and size with clumped chromatin hyperchromatic (↑) or with pyknotic nuclei (red ↑). Striated duct (D) appears disorderly arranged epithelium lining with loss of basal striation. Execratory ducts (*) appear with loss of pseudo-stratification and stagnated secretion in the lumen. The duct was surrounded by dens fibrous connective tissue (CT) with hyalinization area (h) and congested dilated blood vessels (bifid head). Inset hyper magnification of straited duct (D) (HE; D × 100, E × 200, F × 400; inset × 400). Low dose of PIC (PIC-1 group) (**G**–**I**) showing improvement in the histological structure. Some areas of serous acini (A) contain large hyperchromatic nuclei (↑). Apparently large lobules are separated by relatively thin connective tissue septa (s) with few collagens’ fibers deposition. Striated duct (D) with columnar cells and basal striation is detected. Some of the excretory ducts (*) shows loss of pseudo-stratification in some areas. (HE; G × 100, H × 200, I × 400; inset × 400). High dose of PIC (PIC-2 group) (**J**–**L**) showing marked improvement in the histological structure. The parotid gland appears nearly as the control group. Each lobule is formed of serous acini (A) and ducts (D) with wide connective tissue septa filled with a fine network of interlobular connective tissue fibers (CT) serous acini (A) consists of a single layer of pyramidal cells (↑) with basal rounded nuclei surrounding a lumen. Among the acini, striated ducts (D) are observed and lined by a single layer of columnar epithelium (dot arrow). (HE; J × 100, K × 200, L × 400; inset × 400).

HE-stained sections of the parotid gland of the control group showed regular histological features of parenchymal elements. As each lobule of the gland had its own set of serous secretory acini and connective tissue, the land was divided into smaller sections by the interlobular connective tissue. There was just one layer of pyramidal cells with round nuclei surrounding a narrow lumen of each serous acini. Striated ducts appeared intervening between the acini, lined by a single layer of columnar epithelium with centrally placed nuclei, eosinophilic cytoplasm, and basal eosinophilic striations. An intercalated duct surrounded by cells with a basophilic cytoplasm and rounded nuclei located in the middle (Figure 2A–C).For ISO treated rats’ group (Figure 2D–F), the parotid acini appeared markedly hypertrophic and packed together. Some of the acini exhibited irregular outlines, and spaces appeared between acini. Relatively thin connective tissue septa separated large lobules. The cytoplasm and basal line blurred, and acinar cells had evident vacuolization. The nuclei appeared enlarged and irregular in shape and size with clumped chromatin.

Some of the acini showed deeply basophilic stained pyknotic nuclei. Aside from that, cells in the gland ducts were distributed in an unorganized fashion with the lack of basal striation in the striated duct, which had smaller secretory granules. In addition, the excretory ducts showed loss of pseudo-stratification with flattening of the cells and stagnated secretion in the lumen. The duct was surrounded by dense fibrous connective tissue with a hyalinization area and congested dilated blood vessels. In the current study, the parotid gland of a low dose of PIC (PIC-1 group) revealed an improvement in the histological structure. The parotid gland showed an almost normal appearance in most serous acini and ductal systems except for some areas of serous acini containing large hyperchromatic and pleomorphic nuclei. A striated duct with columnar cells and basal striation was detected. A few excretory ducts revealed a loss of pseudo-stratification in some areas. Relatively thin connective tissue septa separate large lobules with few collagen fibers deposition (Figure 2G–I). Interestingly, a high dose of PIC-treated rats (PIC-2 group) revealed marked improvement in the histological structure. The parotid gland appeared nearly as the control group (Figure 2J–L). Each lobule is formed of serous acini and ducts with wide connective tissue septa filled with a fine network of interlobular connective tissue fibers, serous acini consists of a single layer of pyramidal cells with basal rounded nuclei surrounding a lumen. Among the acini, striated ducts were observed and lined by a single layer of columnar epithelium.

**Sublingual Gland**

**Figure 3:**
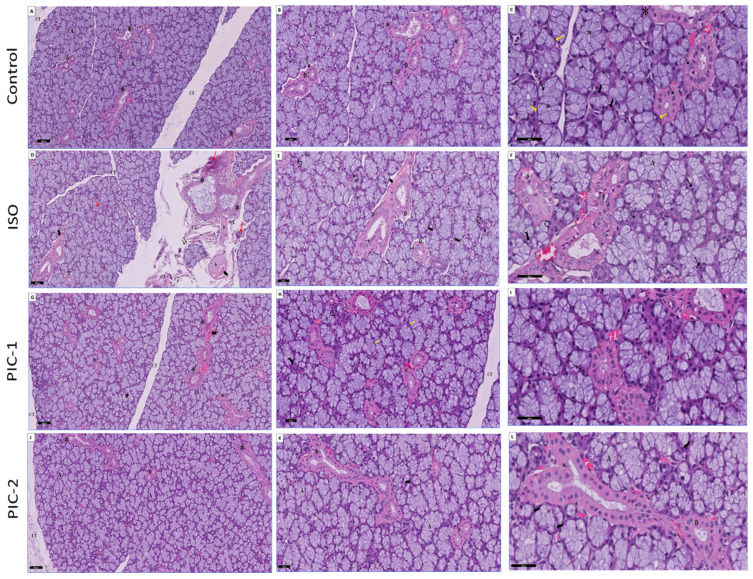
A section in the sublingual gland stained with HE from the control group (**A**–**C**) showing sublingual gland mucous acini and ducts appear with a fine network of interlobular connective tissue (CT). Each mucous acinus (A) consisted of large pyramidal mucous cells with abundant pale blue vacuolated cytoplasm. Acinar mucous cells contain flattened basal nuclei and pale eosinophilic cytoplasm (↑). A serous demilune is seen at the left of the acinus with rounded nuclei, and basal dark linear stain (wavy arrow). The striated duct is lined by columnar cells having eosinophilic cytoplasm and prominent cytoplasmic striations (dot arrow). The excretory ducts (*) have tall columnar epithelium with more apically located nuclei. Myoepithelial cells (yellow arrow) show a narrow cytoplasm and a very flattened nucleus, may be found wrapping ducts and some acini. (HE; A × 100, B × 200, C × 400). ISO group (**D**–**F**) showing markedly hypertrophic packed secretory mucous acini (A) with some areas with shrunken (curved arrow) loss of the acini (↑↑). A serous demilune is seen with vacuole-like structures (wavy arrow). Striated duct (D) appears disorderly arranged epithelium lining with loss of basal striation and marked vacuolation (dot arrow). Execratory ducts (*) appear with loss of pseudo-stratification and stagnated secretion in the lumen. Notice numerous mononuclear cellular infiltration (red arrow), hyalinization area (h), and dilated congested blood vessels (bifid arrow) in the connective tissue septa. (HE; D × 100, E × 200, F × 400; inset × 400). Low dose of PIC (PIC-1 group) (**G**–**I**) showing almost mucous acini (A) and striated ducts (D) appear nearly as control with a fine network of interlobular connective tissue (CT). A serous demilune at the left of the acinus (wavy arrow) and myoepithelial cells (yellow arrow) are seen. Excretory ducts (*) are possessed normal appearance of cell lining and are filled with a small amount of secretion. Notice few mononuclear cellular infiltrations (red arrow) and mild congested blood vessels (bifid arrow) (HE; G × 100, H × 200, I × 400; inset × 400). High dose of PIC (PIC-2 group) (**J**–**L**) showing marked improvement in the histological structure. The sublingual gland appears nearly as the control group. (HE; J × 100, K × 200, L × 400; inset × 400).

HE-stained sections of the sublingual gland of the control group showed sublingual gland mucous acini and ducts with a fine network of interlobular connective tissue. Each mucous acinus consisted of large pyramidal mucous cells with abundant pale blue vacuolated cytoplasm. Acinar mucous cells contain flattened basal nuclei and pale eosinophilic cytoplasm. Mucous acini were mainly included with sets of serous cells, which looked like crescents at the edges of the mucous acini (crescents or demilunes of Gianuzzi or Von Ebner). Myoepithelial cells showed a narrow cytoplasm and a very flattened nucleus, which could be found wrapping excretory ducts and some acini (Figure 3A–C).

Structural alterations in the acini and ducts of ISO-treated rats were visible in HE-stained sections. The sublingual gland showed markedly hypertrophic packed secretory mucous acini with some areas with shrunken and areas with loss of the acini. The nuclei appeared enlarged and irregular in shape and size with clumped chromatin. A serious demilune was seen with vacuole-like structures. Cytoplasmic vacuoles were seen in the cells of the striated ducts, and the epithelial lining had lost its basal striation. Execratory ducts appeared with a loss of pseudo-stratification and stagnated secretion in the lumen. At the same time, certain acini were reduced in size, and the invading mononuclear cells filled in the voids. The lining epithelial cells of certain ducts were destroyed, significantly affecting those ducts. Mononuclear cells infiltrated the interlobular connective tissue septa, hyalinization, and dilated congested blood vessels were seen. Excessive vacuolation of the lining epithelial cells of some excretory ducts was seen, whereas the epithelium of others had been destroyed. Execratory ducts were found to have stagnant secretions in their lumens.

Microscopical images of the submandibular gland in the PIC-1 group (low dosage) showed significant improvements in acini and duct lining cells, and the acini’s morphology was generally intact (Figure 3G–I). The sublingual gland showed almost normal mucous acini and striated ducts. Excretory ducts possessed cell lining with a pseudo-stratified columnar epithelial appearance. Most duct lumens were normal or filled with a small amount of secretion.

Interestingly, a high dose of PIC-treated rats (PIC-2 group) revealed marked improvement in the histological structure. The sublingual gland appeared similar to the control group (Figure 3J–L). Sublingual gland mucous acini and ducts appeared with a fine network of interlobular connective tissue. Many intralobular acini and ducts were more or less as a control group. Mucous acini were mainly included with sets of serous cells. Myoepithelial cells showed a narrow cytoplasm and a very flattened nucleus, which could be found wrapping excretory ducts and some acini.

**Submandibular Gland**

**Figure 4:**
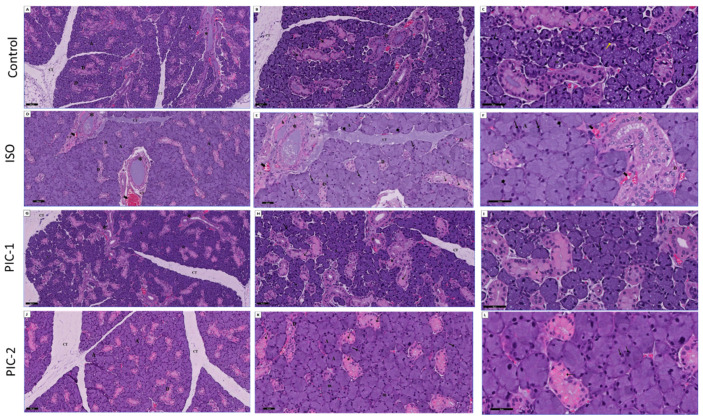
A section in the submandibular gland stained with HE from the control group (**A**–**C**) showing numerous lobules separated by thin connective tissue septa (CT). The lobules show closely packed mixed secretory acini (A), granular convoluted tubules (D) and execratory ducts (*). The acinar cells are mixtures of serous (↑) and mucous (m) secretory cells. The myoepithelial cells (yellow arrow) appear gasping the acini. The striated ducts are lined by columnar cells having oval nuclei (D). The well-developed granular convoluted duct is lined by columnar cells having eosinophilic cytoplasm (dot arrow). The excretory ducts (*) have tall columnar epithelium with more apically located nuclei and prominent cytoplasmic striations. (HE; A × 100, B × 200, C × 400). ISO group (**D**–**F**) showing markedly hypertrophic packed secretory acini (A). The normal architecture of acinar cells is distorted, their nuclei became dark and deeply stained atrophied nuclei (arrowhead). The cytoplasm lost its basophilic character (A). Giant nuclei (↑) are also seen. The cytoplasmic vacuolation in ductal (D) cells were increased with reduced acidophilic content of the cytoplasm of granular duct cells (dot arrow). Execratory ducts (*) appear with loss of pseudo-stratification and stagnated secretion in the lumen. The duct was surrounded by dens fibrous connective tissue (CT) with hyalinization area (h) and congested dilated blood vessels (bifid arrow). (HE; D × 100, E × 200, F × 400). Low dose of PIC (PIC-1 group) (**G**–**I**) showing marked improvement in the histological structure of cells of acini (A) as well as cells of ducts lining, and the acini relatively preserve their shape are seen. The numbers of vacuoles decrease, and well-formed striated ducts (D) are also detected. The granular convoluted tubules were lined by simple columnar epithelium with eosinophilic cytoplasm and basal rounded nuclei. (HE; G × 100, H × 200, I × 400). High dose of PIC (PIC-2 group) (**J**–**L**) showing marked improvement in the histological structure. The submandibular gland appears nearly as the control group. (HE; J × 100, K × 200, L × 40).

Submandibular salivary gland histology revealed parenchymal tissue and connective tissue stroma as the main structural components in control group rats. The secretory acini were numerous, rounded in shape, small, and exhibited narrow lumen. It contains both serous and mucous secretory cells in the acini. Deep basophilic staining was seen in the basilar part of the cell close to its nucleus, indicating that it was a pyramidal cell. In the cell’s apical region, secretory granules can be found. Mucin droplets in mucous acinar cells appear as distinct vacuoles in the apical cytoplasm. The nuclei of these organisms were spherical. The secretory acini were surrounded by myoepithelial cells. Intercalated ducts, granular convoluted tubules, striated ducts, and excretory ducts make up the duct system. Columnar cells with vesicular nuclei and strong eosinophilic cytoplasm bordered the striated ducts, which were visible. Accurately produced convolutions were bordered by epithelial cells with acidic granular cytoplasm and basally located nuclei in the high-columnar epithelium (Figure 4A–C).

Submandibular gland histological degenerative changes were seen in the ISO-treated rats’ group (Figure 4D–F). Hypertrophied acini and distended, conspicuous cells were visible in sections stained with HE. Disrupted acinar cell architecture resulted in dark and discolored nuclei. The cytoplasm was no longer basophilic. Various cell diameters (anisocytosis) and different nuclear sizes were found in the acinar cells (anisonucleosis). There were also large nuclei, and prominent blood capillary congestion could be detected. Vacuolation in ductal cells rose when acidophilic content in granular cells was decreased (Figure 3D–F).

In the present study, the submandibular gland of a low dose of PIC (PIC-1 group) revealed an improvement in the histological structure. The acini and duct lining cells showed significant improvement in the microscopical image, and the acini retained their morphology well. The number of vacuoles was reduced, as well as well-formed striated ducts. Baselines were restored, and the epithelium lining was intact. Between the acini, intercalated ducts could be seen. Eosinophilic cytoplasm and nuclei with basal spherical shapes lined the granular convoluted tubules. No congestion or regions of bleeding were found surrounding these ducts.

Interestingly, a high dose of PIC-treated rats (PIC-2 group) revealed marked improvement in the histological structure. The submandibular gland appeared similar to the control group (Figure 2J–L). Submandibular lobules showed closely packed mixed secretory acini, granular convoluted tubules and execratory ducts. The striated ducts are lined by columnar cells having oval nuclei. The well-developed granular convoluted duct is lined by columnar cells having eosinophilic cytoplasm. The excretory ducts have tall columnar epithelium with more apically located nuclei and prominent cytoplasmic striations.

#### 3.1.2. Examination of Masson Trichrome-Stained Sections

**Figure 5:**
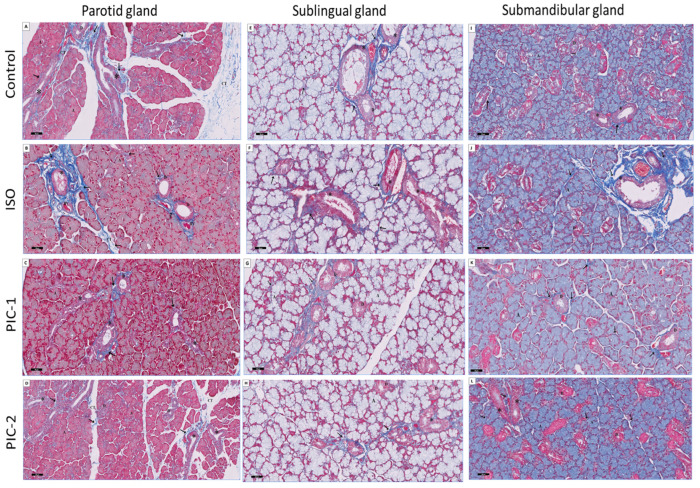
Masson trichrome-stained sections of the three salivary glands (parotid (**A**–**D**), sublingual (**E**–**H**), and submandibular (**I**–**L**) glands) of: control group showing scanty collagen fibers in the connective tissue septa between the lobules surrounding the blood vessels and large excretory ducts (*) are seen. The ISO treated group shows marked deposition of abundant collagen fibers in the connective tissue septa between the lobules that extended to surround the intralobular secretory acini and ducts. The PIC-1 group shows an apparent decrease of collagen fibers deposition in connective tissue septa between the lobules and around the intralobular acini and ducts. The PIC-2 group reveals scanty collagen fibers and appeared similar to the control group. A: acini, D: ducts, CT: connective tissue. (Masson trichrome; ×200).

Examination of Masson trichrome-stained sections of the three salivary glands (parotid, sublingual, and submandibular glands) of the control group revealed scanty collagen fibers in the connective tissue septa between the lobules surrounding the blood vessels and large excretory ducts. Conversely, the ISO treated group revealed marked deposition of abundant collagen fibers in the connective tissue septa between the lobules that extended to surround the intralobular secretory acini and ducts. PIC-1 group also demonstrated a reduction in collagen fiber deposition in connective tissue septa between lobules and surrounding the intralobular ducts and acini. Interestingly, the PIC-2 group revealed scanty collagen fibers and appeared similar to the control group (Figure 5).

### 3.2. Histochemical Results

#### 3.2.1. Examination of Alcian Blue Stain-Stained Sections

**Figure 6:**
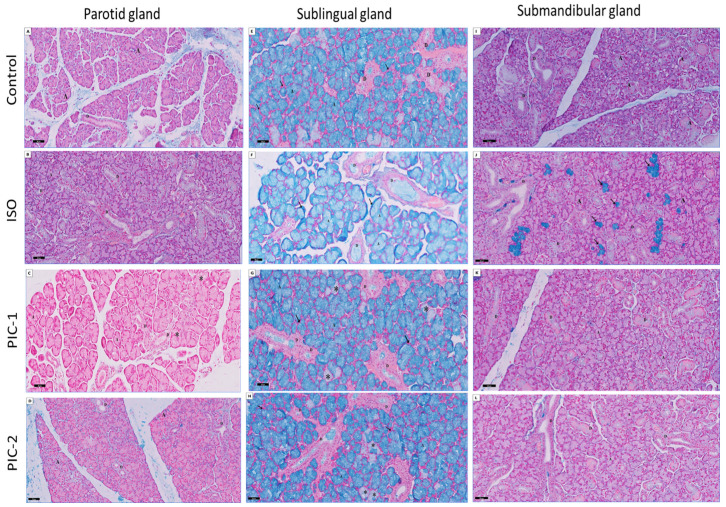
Sections of the three salivary glands (parotid (**A**–**D**), sublingual (**E**–**H**), and submandibular (**I**–**L**) glands) are stained with Alcian blue for acidic glycoconjugates and counterstained with Nuclear Fast red. Control group strong positive acidic mucin sky-blue reaction in the secretory acini (A) and the lumen of ducts (D) in the sublingual gland with a negative reaction in the serous demilune (↑) and negative reaction in the parotid gland. While submandibular gland showed a negative reaction with faint sky-blue color inside the acinar cells. The ISO treated group shows a faint positive acidic mucin sky-blue reaction in the sublingual secretory acini and the lumen of ducts and a strong positive reaction in the serous demilune. The parotid gland shows a negative reaction with faint sky-blue color inside the acinar cells. The submandibular gland shows a blue positive reaction (↑) in the acinar cell’s cytoplasm. The duct epithelial cells show a strong positive reaction for the secretory granules and the epithelial lining. The PIC-1 group shows strong positive acidic mucin sky-blue reaction except for few acinar cells with faint (*) positive reaction. A negative reaction is shown in most of the acini and ducts of the parotid gland while submandibular gland shows negative reaction in most of the acinar cells. The PIC-2 group appears similar to the control group (Alcian blue; ×200).

Examination of Alcian blue-stained sections of the sublingual gland of the control group revealed that the mucous cells of the sublingual gland exhibited a strong and positive staining sky-blue stain, while the serous cell of the parotid gland did not. Sulphated mucopolysaccharides are likely to be abundant in demilunar cells. Strong positive acidic mucin sky-blue reaction in the secretory acini and the lumen of ducts and negative reaction in the parotid gland were noticed. In comparison, the submandibular gland showed a negative reaction with a faint sky-blue color inside the acinar cells. The strong purple staining indicates that most of the submandibular gland’s acinar cells contain a combination of neutral and acid polysaccharides. The ISO treated group revealed a faint positive acidic mucin sky-blue reaction in the secretory acini and the lumen of ducts and a strong positive reaction in the serous demilune. At the same time, the parotid gland showed a negative reaction with the faint sky-blue color inside the acinar cells. Moreover, the submandibular gland showed some of the acinar cell’s cytoplasm, revealing a blue positive reaction for Alcian blue stain. The duct epithelial cells showed a strong positive reaction for the secretory granules and the epithelial lining. In addition, the PIC-1 group showed a strong positive acidic mucin sky-blue reaction in the secretory acini and the lumen of ducts except for a few acinar cells with faint positive reactions. Negative reaction in most of the acini and ducts of the parotid gland with few faint positive acidic mucins’ blue reactions in the acini. In contrast, the submandibular gland showed a negative reaction in most acinar cells. Interestingly, the PIC-2 group appeared nearly as a control group (Figure 6).

#### 3.2.2. Examination of PAS-Stained Sections

Generally, glycoprotein and glycogen are primarily indicated by an intense magenta hue in the PAS-positive response. PAS reactions were seen in only ducts and acini of the parotid and submandibular salivary glands of control groups and in low and high dosage PIC-1, and PIC-2 treated groups, respectively. The PAS staining technique revealed that the serous acini of the parotid gland exhibited strong and positive staining magenta-red color stain. In contrast, the sublingual gland’s secretory acini showed the mucous acini’s pale magenta color, indicating a positive reaction to their neutral and acidic mucin. Moreover, the secretory acini of the submandibular gland revealed a positive reaction (magenta color) toward PAS, indicating the absence of non-sulfated acidic mucin in their mucous acini. However, ISO treated group revealed a faint positive PAS reaction in both the acini and the ducts.

**Figure 7:**
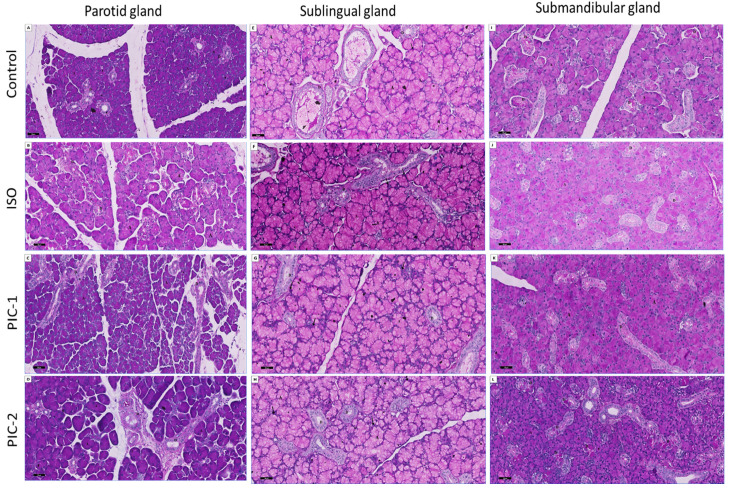
Sections of the three salivary glands (parotid (**A**–**D**), sublingual (**E**–**H**), and submandibular (**I**–**L**) glands) are stained with Alcian blue for Periodic Acid-Schiff (PAS) for identification of neutral mucin and counterstained with Hematoxylin. Sections in the parotid and submandibular salivary gland of control groups and in the low dose PIC-1 and high dose PIC-2 treated groups show strong positive PAS reaction (magenta color), appear in both ducts (D) and acini (A) which is observed more at their basement membrane. However, ISO treated group reveal faint positive PAS reaction in both the acini and the ducts. Most of acinar cells of the sublingual gland of control group exhibit faint positive magenta color reaction in the secretory acini (A) and the lumen of ducts (D) and strong positive reaction in the demilunar cells (↑). However, the ISO treated group shows a strong positive reaction in the secretory acini and the lumen of ducts. Demilunar cells show evident faint positive reaction. Interestingly, low dose PIC-1 and high dose PIC-2 appear nearly as control group compared to the previous groups. The duct cells show a slight increase in the intensity of PAS compared to the ISO treated group (PAS; ×200).

Regarding histochemistry, most of the acinar cells of the sublingual gland of the control group revealed a faint positive magenta color reaction in the secretory acini and the lumen of ducts and a strong positive reaction in the demilunar cells. However, ISO treated group revealed a strong positive reaction in the secretory acini and the lumen of ducts. In addition, sulphated mucopolysaccharides were found to have increased in demilunar cells. Interestingly, low dose PIC-1 and high dose PIC-2 appeared nearly as control groups compared to the previous groups. Compared to the ISO-treated group, the duct cells showed a slight increase in the intensity of PAS (Figure 7).

### 3.3. Immunohistochemical Results

**Figure 8:**
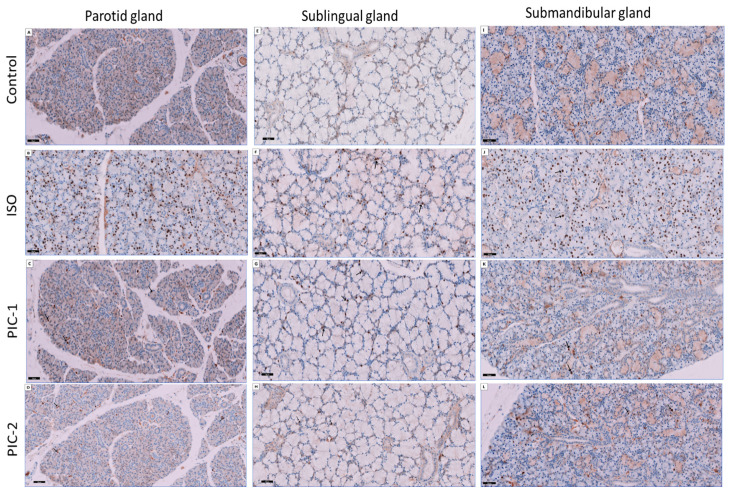
Sections of the three salivary glands (parotid (**A**–**D**), sublingual (**E**–**H**), and submandibular (**I**–**L**) glands) are stained. The control group shows few immunopositive staining to Ki-67 in the secretory acini and ducts. The ISO treated group shows marked increased numbers of immunopositive labeled nuclear staining in the secretory acini. The PIC-1 group shows an apparent decrease in the numbers of immunopositively labeled nuclear staining in the secretory acini and ducts as compared to the ISO group. The PIC-2 group reveal few immunopositive staining to Ki-67 in the secretory acini and ducts and appeared similar to the control group (immunohistochemical stain Ki-67; ×200).

The parotid, sublingual, and submandibular salivary glands (Figure 8) of rat specimens obtained from control rats revealed few immunopositive labeled nuclear staining in the acini, striated, and excretory ducts. The parotid, sublingual, and submandibular salivary gland specimens from the ISO treated rats group revealed marked increased immunopositivity labeled nuclear staining in the secretory acini. The PIC-1 and PIC-2 groups displayed minor positive nuclear staining of Ki-67 antibody tagged nuclei of acinar and negative ductal cells compared to the ISO group, which had no such findings. This might indicate attempts at regeneration and proliferation by the intact parenchymal and ductal cells.

## 4. Morphometric and Statistical Results

### 4.1. Area %age of Collagen Fibers

**Figure 9:**
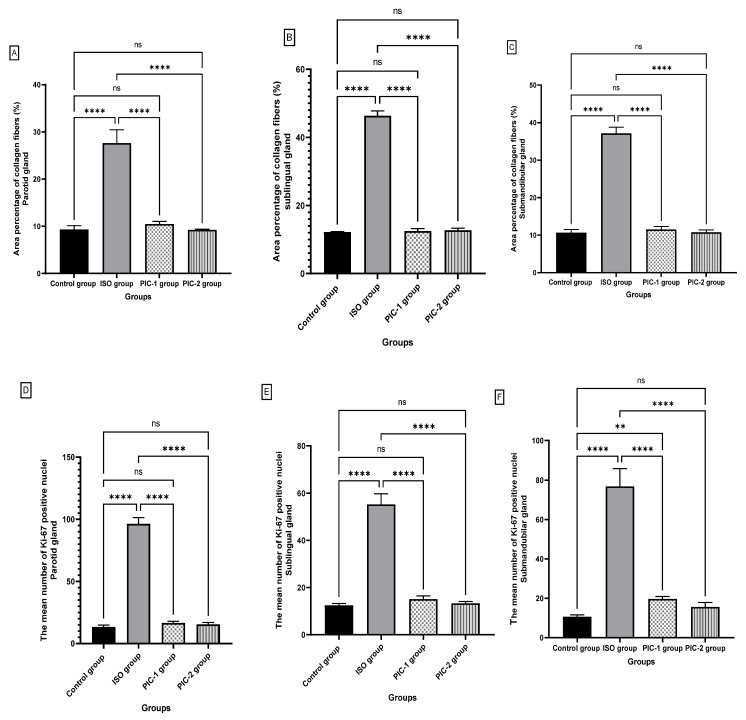
Graph of the mean area %age of collagen fibers (%) in the three salivary glands (parotid (**A**), sublingual (**B**), and submandibular (**C**) glands). The mean number of Ki-67 positive nuclei in the three salivary glands (parotid (**D**), sublingual (**E**), and submandibular (**F**) glands). Data are presented as mean ± SD. Differences between groups were identified using one-way ANOVA, followed by Tukey’s multiple comparison post-hock-test, indicated above the bars. (*n* = 8 rat/group). ** *p* ≤ 0.01, **** *p* ≤ 0.00001, ns = non-significant to the corresponding group.

The mean area %ages of collagen fibers are shown in Figure 9A–C in the three salivary glands (parotid, sublingual, and submandibular glands) of the control group (9.32 ± 0.80), (12.20 ± 0.12), (10.70 ± 0.79), respectively. The greatest mean area %age of collagen fibers was recorded in the ISO group (27.64 ± 2.83), (46.39 ± 1.42), (37.14 ± 1.62); respectively. Using Tukey’s post hoc test, we found a statistically significant difference (*p* ≤ 0.0001). Interestingly, PIC treatment with low dose (PIC-1) (10.47 ± 0.58), (12.51 ± 0.71), (11.51 ± 0.76); respectively, and high dose (PIC-2) (9.26 ± 0.12), (12.76 ± 0.57), (10.76 ± 0.62), respectively, revealed a significant decrease (*p* ≤ 0.0001) in the mean area %age of collagen fibers in the three salivary glands (parotid, sublingual, and submandibular glands) as compared to the ISO group. Moreover, there was a non-significant difference between the PIC-1 and PIC-2 treated groups in the three salivary glands: parotid (*p* = 0.52, *p* > 0.99), sublingual (*p* = 0.95, *p* = 0.66), and submandibular glands (*p* = 0.4862, *p* > 0.99) in contrast with the control group.

### 4.2. Ki-67 Immunoexpression

In the three salivary glands (parotid, sublingual, and submandibular), the mean number of Ki-67 positive nuclei was (13.38 ± 1.40), (12.50 ± 0.75), and (10.63 ± 0.91), respectively, in the control group (Figure 9D–F). The ISO group had the highest mean of Ki-67 positive nuclei (96.38 ± 4.984), (55.25 ± 4.528), (76.75 ± 9.08), respectively. Using Tukey’s post hoc test, we found a statistically significant difference (*p* ≤ 0.0001). Interestingly, PIC treatment with low dose (PIC-1) (16.63 ± 1.18), (15.13 ± 1.35), (19.75 ± 1.16), respectively, and high dose (PIC-2) (15.50 ± 1.51), (13.38 ± 0.74), (15.63 ± 2.26), respectively, revealed a significant decrease (*p* ≤ 0.0001) in the three salivary glands in contrast with the ISO group. Moreover, there was a non-significant difference between the PIC-1 and PIC-2 treated groups in the parotid (*p* = 0.12, *p* = 0.51) and sublingual (*p* = 0.17, *p* = 0.96) compared to the control group. However, submandibular glands revealed a non-significant (*p* = 0.20) difference between PIC-2 and the control group and a significant difference between PIC-1 (*p* ≤ 0.001) and the control group.

## 5. Discussion

The salivary glands have grown to be a valuable tool in studying of basic pharmacological questions [42]. Mouse parotid and sublingual glands produce saliva that facilitates proper mouth function by generating of serous and mucous-type acinar cells. Major acinar cells shift and differentiate during salivary gland formation [43].

A vital point to note is that parotid glands in humans have a serous profile responsible for most stimulation of saliva flow. On the other hand, the sublingual gland produces mucous, while the submandibular gland forms a mixed gland that produces 60% of unstimulated saliva. Human parotid and sublingual glands have similar properties to the serous profile of submandibular glands, which can release growth factors through ducts. Granular convoluted tubules in mouse submandibular glands are unique to rodents and can emit physiologically active polypeptides with local and systemic activities [44,45].

There is currently no effective treatment for the loss of oral and overall health caused by the degeneration of the salivary glands and hyposalivation; however, these problems are significant and related to various physiological conditions, including radiation therapy for head and neck cancer, Sjögren’s syndrome, and old age [46,47,48].

Multiple studies have demonstrated the effectiveness of the non-selective β agonist ISO on salivary glands. In general, gland enlargement observed in the salivary gland after administration of isoproterenol is due to hyperplasia and hypertrophy [49,50,51]. In addition, non-selective agonist isoproterenol treatment in salivary gland tissue significantly reduces saliva production due to the low mitotic rate [17].

Consequently, a method that facilitates the regeneration of salivary glands and restores saliva production is urgently needed. In addition, plant-based drugs are becoming increasingly popular for preventing and treating various pathological conditions worldwide. The current study sheds light on the histopathological changes by which PIC alleviates the degenerative changes by ISO.

The histological changes in the three main salivary glands in the ISO salivary gland dysfunction rat model were examined in the current study. According to our findings, ISO submandibular and sublingual salivary glands appeared to have structural problems. According to the results of this study, ISO injection in rats caused degenerative structural changes in the tissue of the three salivary glands. Multiple cytoplasmic vacuolizations were seen in the secretory acini, markedly hypertrophic and packed. In addition, some of the acini exhibited irregular outlines with inflammatory cell infiltration. Chronic administration of ISO is associated with excessive activation of protein synthesis, enlargement of acinar cells, hyperplasia, and salivary gland enlargement in both humans and rats [52,53]. Similarly, Hagen et al. [51] show that the acinar cells of the parotid gland are markedly hypertrophic and hyperplastic when isoproterenol is administered systemically. In addition, enlarged glandular tissues are associated with significant cell alterations that increase glandular protein production and modifications to the RNA transcription process.

In addition, the ISO group has shown cytoplasmic vacuoles in the parotid gland secretory acini [54]. This was found by Elkhier and his colleagues [15]. Marked vacuolization was shown in the cytoplasm of the acinar cells of rats feeding on a fluid diet [55,56]. Moreover, the ultrastructure of the irradiated salivary gland showed the presence of intracytoplasmic vacuoles and fusion of several secretory granules [57]. The frequently observed intracytoplasmic vacuolization in the acinar and ductal cells might be caused mainly by mitochondria. This is explained by the fact that sodium ions enter the cells profusely due to the failure of cellular metabolism. This profusion causes an osmotic effect that induces the breakdown of the macromolecules found in the injured cell and the presence of the vacuoles. Moreover, the intracytoplasmic vacuolations have also been explained by the deterioration of other cell organelles, especially the Golgi apparatus, whereby if acquiring a fatty nature, they appear as vacuoles [58]. In addition, the cell vacuoles may be due to reduced secretory activity of the acinar cells and a decrease in the secretory material of the granules [59].

In addition, the excretory ducts in the ISO treated group displayed lumen dilatation, stagnant secretion, and areas of flattening in their epithelial lining. According to Elsharkawy and Alhazzazi, this could be explained, which suggested that this flattening may occur due to the ductal cells’ metaplasia and the accumulated secretion and secondary to glandular injury. Consequently, the resultant impairment of flow rate reflected the glandular dysfunction and xerostomia [60]. Moreover, the apparent presence of dilated congested blood vessels in ISO treated groups and the periductal chronic inflammation could be an inflammatory response to the drug permitting the body to carry more blood to the proliferative areas. In addition, dilatation of blood vessels and their engorgement with RBCs resulting in stasis and a decrease in the blood flow are probably the possible causes of hypoxia and ischemia of the tissues that aggravate the degenerative effects. A decrease in blood flow can cause diminished oxygen and food availability to cells. Toxic metabolites are produced, and cellular energy is reduced because of this process. Reactive oxygen species, such as free radicals, also play a role in the cell damage triggered by the return of blood flow to the ischemic organ [61,62]. Excessive collagen deposition was detected in the ISO group and could be attributed to ISO-mediated increased expression in the activity of matrix metalloproteinases (MMPs) MMP-2 and -9. This results in the development of interstitial inflammation and fibrosis [63].

The histological results of the ISO group were parallel with the histochemical observation of the same group as ISO led to a decrease in both acid and neutral mucosubstances while there is an increase in acid mucosubstances demilune cells. In contrast, the parotid gland showed decreased acidic mucin. Moreover, the submandibular gland showed some of the acinar cell’s cytoplasm revealed areas with increased acidic mucin. The duct epithelial cells showed a strong positive reaction for the secretory granules and the epithelial lining. The alteration of secretory granules within the cells after they failed to discharge due to a lack of stimulation might explain the change in histochemistry of secretory granules [64,65,66].

The striated ducts were affected by ISO. Loss of basal striation was seen, along with vacuolation and degenerative changes. Striated ducts play a role in regulating the salivary secretion produced by secretory units. It has been reported that they reabsorb electrolytes and organic materials from primary saliva. They can also synthesize and secret glycoprotein into saliva [64].

During the S, G2, and M stages of mitosis, Ki67 staining is widely used as a proliferation indicator. In addition, regardless of the cause for entering G0/quiescence, Ki67 continues to degrade in the G1 and G0 phases. When it comes to individual cells, it is variable and reliant on how long the cell has been in G0 [67].

There were few positive nuclear reactions to Ki-67 in the control group in the current study and a significant increase in the treated rats’ group. Isoproterenol causes significant protein release [68,69] from the parotid glands of mice and rats and glandular hypertrophy when administered systemically [70,71,72]. Both hyperplasia (increased acinar cell counts) and hypertrophy (increased acinar cell size) are present in this glandular growth (increased size of acinar cells). DNA synthesis commences within 24 h of isoproterenol injection, with peak mitotic activity happening 35 h later. DNA synthesis and mitotic activity inside the parotid gland have been proven to rise in tandem with the increased glandular protein production [51].

Similarly, Katsumata and his coworkers [73] found that ISO treated rat parotid glands’ acinar cells showed higher proliferation on day three of ISO. Acini enlargement and an increase in the mean number of proliferating cells were seen following two days of salbutamol (selective adrenoceptors agonist) therapy, according to Metwally and his coworkers [74]. In addition, the proliferation of ducts and specific endothelial cells was boosted. After 4–6 days of therapy, the proliferative activity was most abundant and remained significant after ten days of treatment with ISO [74]. Another study evaluated histomorphometry age-related changes in aging rats’ submandibular and sublingual salivary glands. It concluded a marked increase in the mean number of Ki-67 positive cells in seromucous acinar cells accompanied by a lower decrease in mucous acinar cells [75]. These findings correlate with our study’s findings as Ki-67 expression was significantly higher in parotid and submandibular than sublingual gland.

Plants create polyphenolic stilbenes, resveratrol, and piceatannol in reaction to fungal infection, mechanical injury, or ultraviolet irradiation. Since they have no adverse effects on the body, they can be consumed regularly. Their contributions significantly improve human health. Precisely because of its antioxidant effect, piceatannol outperforms resveratrol in terms of anticancer efficacy [76].

Interestingly, no study has detected the potential protective effect of PIC after ISO treatment on the salivary gland. However, there has been a different study on the protective effect of resveratrol in salivary gland dysfunction [32,33,34].

In the present study, the acinar and ductal cells of the three salivary glands for the low dose PIC-1 treated groups presented fewer atrophic changes with a partial absence of intracytoplasmic vacuolization. The cytoplasm was probably protected from the damaging effect of ISO, while in a high dose of PIC-2 treated group, the acinar and duct cells presented similar to the control group. There was an apparent change regarding histochemical observations, and it appeared similar to the control group. Kim et al. [77] suggested the possibility of using polydatin (resveratrol) as a therapeutic drug to improve hyposalivation caused by diabetes. AQP5 overexpression and polydatin’s antioxidant and anti-glycation actions boosted mucin accumulation, and they found that polydatin had a significant protective impact on diabetes-related salivary gland hypofunction. Xerostomia is irradiation’s common acute adverse effect, caused by direct ionization damage to salivary gland cells. This natural antioxidant may help reduce the harmful effects of irradiation-induced salivary gland dysfunction. Resveratrol at relatively high dosages reduces irradiation-dependent salivary gland damage [78]. Inoue and colleagues verified the swelling of the NOD mice’s salivary glands. Furthermore, salivary glands in the resveratrol-administered group have shown increased IL-10 expression. Because of this, they found a unique therapeutic benefit for Sjögren’s syndrome salivary dysfunction resveratrol [33].

The infiltration of inflammatory cells was shown to be reduced in PIC in the current investigation. Anti-inflammatory pathways may be activated by piceatannol, which may help decrease inflammation. Piceatannol activated the transcriptional activity of nuclear factor erythroid 2-related factor 2 (Nrf2) in human endothelial cells, increasing the expression of the antioxidant and anti-inflammatory enzyme heme oxygenase-1 (HO-1). This increase was concentration-dependent. When HO-1 was inhibited, piceatannol lost its ability to suppress TNF-, IL-6, and NF-B transcriptional activity and phosphorylation of p65 [27].

Collagen fiber deposition and Ki-67 immunohistochemistry positive nuclear cells were significantly reduced in this research. In addition, Ki-67 positive nuclear cells decreased significantly in animals given piceatannol (12.5 mg/kg) in response to azoxymethane/dextran sulfate sodium-induced colon cancer development [79].

## 6. Conclusions

It is the first time PIC has been shown to reverse histological abnormalities in the salivary glands resulting from exposure to ISO, indicating a possible protective effect. The highest effective dosage was 40 mg/kg, which indicated an anti-ISO effect. Furthermore, PIC reduced Ki-67 expression considerably. PIC’s preventive impact on oxidative, inflammatory, and apoptotic indicators necessitates more research to discover the probable underlying mechanisms for its protective action.

## Data Availability

Data are contained within the article.

## References

[1] Punj A. (2018). Secretions of human salivary gland. Salivary Glands-New Approaches in Diagnostics and Treatment.

[2] Tanasiewicz M., Hildebrandt T., Obersztyn I. (2016). Xerostomia of various etiologies: A review of the litera-ture. Adv. Clin. Exp. Med. Off. Organ Wroclaw Med. Univ..

[3] Glick M. (2015). Burket’s Oral Medicine.

[4] Ngo D.Y.J., Thomson W.M. (2021). An Update on the Lived Experience of Dry Mouth in Sjögren’s Syndrome Patients. Front. Oral Health.

[5] Pedersen A.M.L., Sørensen C.E., Proctor G.B., Carpenter G., Ekström J. (2018). Salivary secretion in health and disease. J. Oral Rehabil..

[6] Pedersen A.M.L., Soerensen C., Proctor G., Carpenter G. (2018). Salivary functions in mastication, taste and textural perception, swallowing and initial digestion. Oral Dis..

[7] Villa A., Connell C.L., Abati S. (2014). Diagnosis and management of xerostomia and hyposalivation. Ther. Clin. Risk Manag..

[8] Smidt D., Torpet L.A., Nauntofte B., Heegaard K.M., Pedersen A.M.L. (2010). Associations between labial and whole salivary flow rates, systemic diseases and medications in a sample of older people. Commun. Dent. Oral Epidemiol..

[9] Fleming M., Craigs C.L., Bennett M.I. (2020). Palliative care assessment of dry mouth: What matters most to patients with advanced disease?. Support Care Cancer.

[10] Tiisanoja A., Syrjälä A.-M., Kullaa A., Ylöstalo P. (2020). Anticholinergic burden and dry mouth in middle-aged people. JDR Clin. Transl. Res..

[11] Lee K.A., Park J.-C., Park Y.K. (2020). Nutrient intakes and medication use in elderly individuals with and without dry mouths. Nutr. Res. Pract..

[12] Pedersen A.M.L., Carpenter G. (2015). Diseases causing oral dryness. Dry Mouth.

[13] Fathi Y., Hoseini E.G., Atoof F., Mottaghi R. (2021). Xerostomia (dry mouth) in patients with COVID-19: A case series. Futur. Virol..

[14] Scully Cbe C. (2003). Drug effects on salivary glands: Dry mouth. Oral Dis..

[15] Abou Elkhier M., Elmeadawy S., Salama N. (2018). Effect of salbutamol on the parotid salivary gland of rats: Ultrastructural study. Egypt Dent. J..

[16] Copik A., Baldys A., Nguyen K., Sahdeo S., Ho H., Kosaka A., Dietrich P.J., Fitch B., Raymond J.R., Ford A.P.D.W. (2015). Isoproterenol acts as a biased agonist of the alpha-1A-adrenoceptor that selectively activates the MAPK/ERK pathway. PLoS ONE.

[17] Susa T., Sawai N., Aoki T., Iizuka-Kogo A., Kogo H., Negishi A., Yokoo S., Takata K., Matsuzaki T. (2013). Effects of repeated administration of pilocarpine and isoproterenol on aquaporin-5 expression in rat salivary glands. Acta Histochem. ET Cytochem..

[18] Alterman A., Mathison R., Coronel C.E., Stroppa M.M., Finkelberg A.B., Gallará R.V. (2012). Functional and proteomic analysis of submandibular saliva in rats exposed to chronic stress by immobilization or constant light. Arch. Oral Biol..

[19] Dhakal B.P., Sweitzer N.K., Indik J.H., Acharya D., William P. (2020). SARS-CoV-2 infection and cardiovascular disease: COVID-19 heart. Heart Lung Circ..

[20] Kochav S.M., Coromilas E., Nalbandian A., Ranard L.S., Gupta A., Chung M.K., Gopinathannair R., Biviano A.B., Garan H., Wan E.Y. (2020). Cardiac arrhythmias in COVID-19 infection. Circ. Arrhythmia Electrophysiol..

[21] Jensen S.B., Pedersen A.M.L., Vissink A., Andersen E., Brown C.G., Davies A.N., Dutilh J., Fulton J.S., Jankovic L., Lopes N.N.F. (2010). A systematic review of salivary gland hypofunction and xerostomia induced by cancer therapies: Prevalence, severity and impact on quality of life. Support. Care Cancer.

[22] Holmberg K.V., Hoffman M.P. (2014). Anatomy, biogenesis and regeneration of salivary glands. Saliva Secret Funct..

[23] Keihanian F., Moohebati M., Saeidinia A., Mohajeri S.A., Madaeni S. (2021). Therapeutic effects of medicinal plants on isoproterenol-induced heart failure in rats. Biomed. Pharmacother..

[24] World Health Organization (WHO) (2018). A Vision for Primary Health Care in the 21st Century: Towards Universal Health Coverage and the Sustainable Development Goals.

[25] Baur J.A., Pearson K.J., Price N.L., Jamieson H.A., Lerin C., Kalra A., Prabhu V.V., Allard J.S., Lopez-Lluch G., Lewis K. (2006). Resveratrol improves health and survival of mice on a high-calorie diet. Nature.

[26] Walle T. (2011). Bioavailability of resveratrol. Ann. N. Y. Acad. Sci..

[27] Kershaw J., Kim K.-H. (2017). The therapeutic potential of piceatannol, a natural stilbene, in metabolic diseases: A review. J. Med. Food.

[28] Setoguchi Y., Oritani Y., Ito R., Inagaki H., Maruki-Uchida H., Ichiyanagi T., Ito T. (2014). Absorption and metabolism of piceatannol in rats. J. Agric. Food Chem..

[29] Eid B.G., Abdel-Naim A.B. (2020). Piceatannol attenuates testosterone-induced benign prostatic hyperplasia in rats by modulation of Nrf2/HO-1/NFκB axis. Front Pharmacol..

[30] Alhakamy N.A., Badr-Eldin S.M., Ahmed O.A.A., Asfour H.Z., Aldawsari H.M., Algandaby M.M., Eid B.G., Abdel-Naim A.B., Awan Z.A., Alghaith A.F. (2020). Piceatannol-loaded emulsomes exhibit enhanced cytostatic and apoptotic activities in colon cancer cells. Antioxidants.

[31] Piotrowska H., Kucinska M., Murias M. (2012). Biological activity of piceatannol: Leaving the shadow of resveratrol. Mutat. Res. Mutat. Res..

[32] Xu L., Yang X., Cai J., Ma J., Cheng H., Zhao K., Yang L., Cao Y., Qin Q., Zhang C. (2013). Resveratrol attenuates radiation-induced salivary gland dysfunction in mice. Laryngoscope.

[33] Inoue H., Kishimoto A., Ushikoshi R., Hasaka A., Takahashi A., Ryo K., Muramatsu T., Ide F., Mishima K., Saito I. (2016). Resveratrol improves salivary dysfunction in a non-obese diabetic (NOD) mouse model of Sjögren’s syndrome. J. Clin. Biochem. Nutr..

[34] Allushi B., Bagavant H., Papinska J., Deshmukh U.S. (2019). Hyperglycemia and salivary gland dysfunction in the non-obese diabetic mouse: Caveats for preclinical studies in Sjögren’s syndrome. Sci. Rep..

[35] Cano I.P., Dionisio T.J., Cestari T.M., Calvo A., Ishikiriama B.L.C., de Faria F.A.C., Siqueira W.L., Santos C.F. (2019). Losartan and isoproterenol promote alterations in the local renin-angiotensin system of rat salivary glands. PLoS ONE.

[36] Wahdan S.A., Azab S.S., Elsherbiny D.A., El-Demerdash E. (2017). Piceatannol ameliorates cisplatin-induced his-tological and biochemical alterations in rats kidney. IJPPS.

[37] Bancroft J.D., Gamble M. (2008). Theory and Practice of Histological Techniques.

[38] Culling C.F.A., Allison R.T., Barr W.T. (1985). Haematoxylin and its counterstain. Cellular Pathology Technique.

[39] Kiernan J.A. (1999). Histological and histochemical methods: Theory and practice. Shock.

[40] Bussari S., Ganvir S.M., Sarode M., Jeergal P.A., Deshmukh A., Srivastava H. (2018). Immunohistochemical detection of proliferative marker Ki-67 in benign and malignant salivary gland tumors. J. Contemp. Dent. Pract..

[41] Fedirko N.V., Kruglikov I.A., Kopach O.V., Vats J.A., Kostyuk P.G., Voitenko N.V. (2006). Changes in functioning of rat submandibular salivary gland under streptozotocin-induced diabetes are associated with altera-tions of Ca^2+^ signaling and Ca^2+^ transporting pumps. Biochim Biophys Acta Mol. Basis Dis..

[42] Al-Refai A.S., Khaleel A.K., Ali S. (2014). The effect of green tea extract on submandibular salivary gland of methotrexate treated albino rats: Immunohistochemical study. J. Cytol. Histol..

[43] Ogawa M., Oshima M., Imamura A., Sekine Y., Ishida K., Yamashita K., Nakajima K., Hirayama M., Tachikawa T., Tsuji T. (2013). Functional salivary gland regeneration by transplantation of a bioengineered organ germ. Nat. Commun..

[44] Porcheri C., Mitsiadis T.A. (2019). Physiology, pathology and regeneration of salivary glands. Cells.

[45] Amano O., Mizobe K., Bando Y., Sakiyama K. (2012). Anatomy and histology of rodent and human major salivary glands—Overview of the Japan salivary gland society-sponsored workshop. Acta Histochem Cytochem..

[46] Atkinson J.C., Grisius M., Massey W. (2005). Salivary Hypofunction and Xerostomia: Diagnosis and Treatment. Dent. Clin. N. Am..

[47] Turner M., Jahangiri L., Ship J.A. (2008). Hyposalivation, xerostomia and the complete denture: A systematic review. J. Am. Dent. Assoc..

[48] Miranda-Rius J., Brunet-Llobet L., Lahor-Soler E., Farré M. (2015). Salivary secretory disorders, inducing drugs, and clinical management. Int. J. Med. Sci..

[49] Hand A.R., Ho B. (1985). Mitosis and hypertrophy of intercalated duct cells and endothelial cells in the iso-proterenol-treated rat parotid gland. J. Dent. Res..

[50] Venkatesh S.G., Tan J., Gorr S.-U., Darling D.S. (2007). Isoproterenol increases sorting of parotid gland cargo proteins to the basolateral pathway. Am. J. Physiol. Physiol..

[51] Ten Hagen K.G., Balys M.M., Tabak L.A., Melvin J.E. (2002). Analysis of isoproterenol-induced changes in parotid gland gene expression. Physiol. Genomics.

[52] Chisholm D.M., Adi M.M. (1995). Cell proliferation and apoptosis in isoprenaline-induced sialosis in the rat submandibular glands. Int. J. Exp. Pathol..

[53] Melvin J.E., Nguyen H.-V., Nehrke K., Schreiner C.M., Hagen K.G.T., Scott W. (2001). Targeted disruption of the Nhe1 gene fails to inhibit β1-adrenergic receptor-induced parotid gland hypertrophy. Am. J. Physiol. Liver Physiol..

[54] Simson J.A.V. (1972). Evidence of cell damage in rat salivary glands after isoproterenol. Anat. Rec..

[55] Leal S.C., Toledo O.A., de Bezerra A.C.B. (2003). Morphological alterations of the parotid gland of rats main-tained on a liquid diet. Braz. Dent. J..

[56] Kuntsal L., Firat D., Sirin Y. (2003). Prevention of liquid-diet-induced damages on submandibular glands by selenium supplementation in rats. Tohoku J. Exp. Med..

[57] Amal A.E.B. (2013). Histological and ultrastructural evaluation of the protective effect of ginseng on gam-ma-irradiated rats’ salivary glands. Nat. Sci..

[58] Al Ankily M., Shamel M., Bakr M.M. (2020). Epidermal growth factor improves the ultrastructure of sub-mandibular salivary glands of streptozotocin induced diabetic rats-a qualitative study. Int. J. Med. Dent. Sci..

[59] Kassab A.A., Tawfik S.M. (2018). Effect of a caffeinated energy drink and its withdrawal on the submandibular salivary gland of adult male albino rats: A histological and immunohistochemical study. Egypt. J. Histol..

[60] Elsharkawy G.E.Z., Alhazzazi T.Y. (2016). The effect of the commonly used antidepressant drug amitriptyline (TCAs) on the salivary glands. J. Dent. Oral Disord. Ther..

[61] Evcimen N.D., King G.L. (2007). The role of protein kinase C activation and the vascular complications of diabetes. Pharmacol. Res..

[62] Moubarak R. (2008). The effect of hypercholesterolemia on the rat parotid salivary gland (histopathological and immunohistochemical study). Cairo Dent. J..

[63] Thangaiyan R., Arjunan S., Govindasamy K., Khan H.A., Alhomida A.S., Prasad N.R. (2020). Galangin attenuates isoproterenol-induced inflammation and fibrosis in the cardiac tissue of albino wistar rats. Front. Pharmacol..

[64] El-Kordy E.A., Alanazi A.D., Ali S.S., Makhlouf M.M.M., Rabah S.O. (2014). Histological, histochemical and uitra-structural changes in the submandibular gland of starved young male cats. J. Cytol Histol..

[65] Al Okaili A.G., Sedeeq B.I., Hazeem M.I. (2009). Histological changes of the submandibular salivary gland of mice maintained on a liquid diet. Tikrit J. Pure Sci..

[66] El-Nozahy A.A., Smail M.I.A. (2013). The response of rat submandibular salivary gland to plant protein diet: Biological and histochemical study. Int. J. Health Sci..

[67] Miller I., Min M., Yang C., Tian C., Gookin S., Carter D., Spencer S.L. (2018). Ki67 is a graded rather than a binary marker of proliferation versus quiescence. Cell Rep..

[68] Berridge M.J. (1982). Regulation of cell secretion: The integrated action of cyclic AMP and calcium. Cyclic Nucleotides.

[69] Albers T., Sabbatini M.E. (2020). Cyclic Nucleotides as Mediators of Acinar and Ductal Function. Pancreapedia Exocrine Pancreas Knowl Base. Pancreapedia Exocrine Pancreas Knowl. Base.

[70] Selye H., Veilleux R., Cantin M. (1961). Excessive stimulation of salivary gland growth by isoproterenol. Science.

[71] Schneyer C.A. (1962). Salivary gland changes after isoproterenol-induced enlargement. Am. J. Physiol. Content.

[72] Thoungseabyoun W., Tachow A., Pakkarato S., Rawangwong A., Krongyut S., Sakaew W., Kondo H., Hipkaeo W. (2017). Immunohistochemical localization of cannabinoid receptor 1 (CB1) in the submandibular gland of mice under normal conditions and when stimulated by isoproterenol or carbachol. Arch. Oral Biol..

[73] Katsumata O., Sato Y.-I., Sakai Y., Yamashina S. (2009). Intercalated duct cells in the rat parotid gland may behave as tissue stem cells. Anat. Sci. Int..

[74] Elhawary Y., Metwally E., Mohammed M.A., Grawish M. (2013). The effect of salbutamol on the parotid salivary gland of albino rats (immunohistochemical study). Res. J. Med. Sci..

[75] Elsaied H.A. (2019). Histological and immunohistochemical study of selenium regenerative effect on submandibular and sublingual glands of aging rats. Egypt Dent. J..

[76] Kukreja A., Wadhwa N., Tiwari A. (2014). Therapeutic Role of Resveratrol and Piceatannol in Disease Prevention. J. Blood Disord. Transfus..

[77] Kim H.R., Jung W.K., Park S.-B., Ryu H.Y., Kim Y.H., Kim J. (2021). Polydatin alleviates diabetes-induced hyposalivation through anti-glycation activity in db/db mouse. Pharmaceutics.

[78] Şimşek G., Gürocak S., Karadaǧ N., Karabulut A.B., Demirtaş E., Karataş E., Pepele E. (2012). Protective effects of resveratrol on salivary gland damage induced by total body irradiation in rats. Laryngoscope.

[79] Kimura Y. (2021). Long-term oral administration of piceatannol (3,5,3′,4′-tetrahydroxystilbene) attenuates colon tumor growth induced by azoxymethane plus dextran sulfate sodium in C57BL/6J mice. Nutr. Cancer.

